# The Effect of Risk Perception on Anxiety in Emerging Adulthood Under the Local Outbreak of COVID-19: A Conditional Process Analysis

**DOI:** 10.3389/fpsyg.2022.759510

**Published:** 2022-03-30

**Authors:** Haojie Fu, Bin Wang

**Affiliations:** ^1^Psychological Education and Counseling Center, Southwest University of Science and Technology, Mianyang, China; ^2^Psychology Department, Southwest University of Science and Technology, Mianyang, China; ^3^School of Humanities and Social Science, University of Science and Technology of China, Hefei, China

**Keywords:** COVID-19, emerging adults, risk perception, anxiety, resilience

## Abstract

This study aims to explore the influence mechanism of COVID-19 risk perception on anxiety in emerging adulthood in the context of public health events of the second round of COVID-19 outbreaks and provide support for exploring the path of mental health after the normalization of the epidemic situation. An online questionnaire, combined with community social work, was used in this study, and data of 522 emerging adults were collected in February 2021. The Perceived Risk of COVID-19 pandemic scale (PRCPS), the generalized anxiety disorder 7-item (GAD-7) scale, the scale of affect balance, and the connor-davidson resilience scale (CD-RISC) were used to investigate. The results showed that: first, the risk perception of COVID-19 in early adulthood was positively predictive of anxiety symptoms [*B* = 0.110, *p* < 0.05, 95%CI = (0.042, 0.176)]. Second, the affective quality of life plays a mediating role between the risk perception of COVID-19 and anxiety [*B* = 0.108, 95%CI = (0.060, 0.161)]. Thirdly, resilience plays a moderating role between the risk perception of COVID-19 and anxiety, the higher the resilience of emerging adulthood, the weaker effects of the risk perception of COVID-19 negative prediction of anxiety [*B* = −0.110, *p* < 0.001, 95%CI = (−0.170, −0.049)]. Therefore, to control the anxiety of emerging adulthood in public health events, we should pay attention to the propaganda and management of epidemic information, improve the quality of life, and attention should be paid to the emerging adulthood with low resilience.

## Introduction

The COVID-19 pandemic, which outbreaks in late 2019 has greatly impacted the normal operation of the world economy and society. The WHO announced the outbreak of COVID-19 as a public health emergency of international concern. In the winter and spring season of 2020–2021, large-scale local outbreaks were detected in the rural areas of Hebei, Jilin, and Heilongjiang provinces, and more than 2,000 cases were reported in total (Gao, 2021). Viral panic, conspiracy ideation, and contagious fear during the COVID-19 pandemic filled everyone’s life and perceived lot of risks (Bratu, 2020; Lăzăroiu and Adams, 2020; Popescu Ljungholm and Olah, 2020). Risk perception in terms of virus anxiety and emotional contagion shaping the COVID-19 pandemic fear (Dobson-Lohman and Potcovaru, 2020; Lăzăroiu et al., 2020; Nemțeanu et al., 2021). In addition to posing a major threat to physical health, the COVID-19 pandemic also poses a threat to mental health due to people’s long-term fear and uncertainty during the epidemic (Adhanom Ghebreyesus, 2020; Hyland et al., 2020). People get depression, anxiety, and psychological stress (Duncan, 2020). And due to the COVID-19, about 24.9% of college students have experienced anxiety (Cao et al., 2020).

Especially for young people, emerging adults are most impaired (Wirkner et al., 2021). According to Arnett (2007), emerging adulthood (18–25 years old) is the age of instability due to residential, love, work, and education changes and a self-focused age because emerging adults have little in the way of social obligations, duties, and commitments to others, which leaves them with a great deal of autonomy in running their own lives. The mental health problems of emerging adults in their early adulthood may be more serious. The uniqueness of early adulthood is that they do not think they are adults or teenagers. Most of them do not have a stable family and living environment (Arnett, 2000). Therefore, emerging adults are one of the groups most may affected by the COVID-19 epidemic, and they have not yet obtained a stable occupational environment and spouse support. When dealing with major public crisis events, the insufficient coping ability of young adults in adulthood will cause mental health problems (Gariepy et al., 2016; Li et al., 2021). A longitudinal study of depression and anxiety among young adults affected by the epidemic in the United States found that they were indeed under great pressure, and the psychological and social stressors brought by the COVID-19 epidemic related to their depression and anxiety (Kujawa et al., 2020).

Risk perception is an individual’s subjective feeling and understanding of external objective stressors, which will be affected by psychological, social, and even cultural factors (Xi et al., 2020). Studies have shown that the perceived risk of the COVID-19 pandemic (PRCP) can positively predict Generalized Anxiety Disorder (GAD; Oyetunji et al., 2021; Yin et al., 2021). But there are also different results, Musche’s study on diabetic patients found that their risk perception is higher than that of the control group, but the level of GAD is no different from that of the control group (Musche et al., 2021). Therefore, PRCP may have a moderating variable in predicting GAD. According to the psychological stress model, combined with stressors, appraisal, social environment, and disorders, which have helped to elucidate the determinants of health in which stress can be an integrative variable (Lemyre and Lalande-Markon, 2009). And Kumpfer and Bluth (2004) resilience framework operationalized resilience as an internal factor which has the characteristic of a dynamic change process by combining the construct internal self-characteristics and resilience processes. Resilience helps reduce the negative impact of adversity on individuals and improve adaptation and growth (Huang et al., 2019). In different individuals, even the stress induced by the same stimulus with different situation will have different effects. Thus, the individual’s resilience factors can help them deal with the negative effects of stress (Wilks and Croom, 2008).

According to the common-sense model (CSM) in risk perception theory, the CSM hypothesizes that individuals create mental representations of their illness based on the concrete and abstract sources of information available to them in order to make sense of and manage the problem. People typically make simultaneous cognitive and emotional representations of their illness (Hagger and Orbell, 2003). Combined with the model of emotion dysregulation proposed by Mennin et al., heightened intensity of emotions, poor understanding of emotions, negative reactivity to emotions, and maladaptive management of emotions—best reflected the structure of four commonly used measures of emotion function and dysregulation, it is believed that the vicious circle mode of emotional dysregulation will bring more avoidance behavior, lead to negative emotional experience and the concurrent symptoms of GAD (Mennin et al., 2007). And emotion can be used as an mediating variable when GAD is affected (Marganska et al., 2013). Therefore, emotional experience may play a mediating role in the model of PRCP affecting GAD.

*H1*: PRCP will have a positive correlation with GAD.

*H2*: Resilience will moderate the effect PRCP has on GAD.

*H3*: The effects of PRCP on GAD will be partially mediated by the IGA.

**Figure 1:**
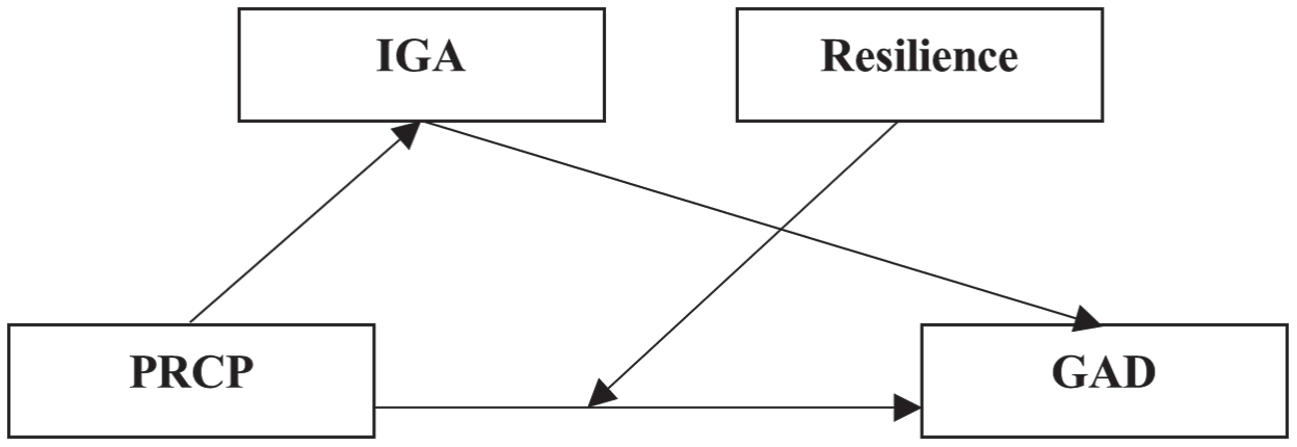
Hypothesized conditional process model.

The overarching aim is to understand the conditional process that GAD is affected by PRCP through IGA, and the extent of GAD affected by PRCP is different for people with different levels of resilience. This study uses the “conditional process model,” a tool for understanding causal processes, to understand the mechanism of independent variables affecting the dependent variable proposed by Hayes (2013). It is used to estimate the direct and indirect pathways through which a variable transmits its effects, as well as to model how the size of those effects depends on (or are conditional on) the value(s) of one or more moderators (Hayes, 2013). We summarize our hypotheses in Figure 1.

## Materials and Methods

### Participants

A total of 704 data were collected in February 2021 in the form of an online questionnaire and combined with community social work in Sichuan Province of China. There is no extensive epidemic in Sichuan, but there have been large-scale local outbreaks in rural areas of other provinces in China, such as Hebei, Jilin and Heilongjiang (Gao, 2021). People perceive the risk of the epidemic, but life has returned to normal. Invalid questionnaires were filtered or according to the following steps: Remove data with an answer time of fewer than 300 s, Find similar (according to IP, submission time, age, residence, etc.) to filter duplicate questionnaires, The Mahalanobis distance is used to exclude data outside the 0.001 standards (Aguinis et al., 2013). A total of 522 valid questionnaires were collected, with an effective rate of 74.15%. The included baseline sample was 37.2% men, 53.1% women, and 9.8% nonbinary. Mean age was 21.77 years (*SD* = 2.26). 7.8% were married, 91.8% were unmarried, and 0.2% divorced.

### Measures

#### Perceived Risk of COVID-19 Pandemic Scale

Perceived Risk of COVID-19 Pandemic Scale was used to assess the PRCP of emerging adults (Xi et al., 2020). The scale consists of nine questions, which are divided into three dimensions: emotional feeling, e.g., I worry about getting infected with COVID-19 (None of the time, Rarely, Some of the time, A moderate amount of time, A lot of the time, All of the time), cognitive judgment, e.g., I am sure I will NOT get infected with COVID-19 (Strongly disagree, Disagree, Somewhat disagree, Somewhat agree, Agree, Strongly agree), and psychological representation of unusual severity, e.g., Getting COVID-19 is something I have (Never thought about, Rarely thought about, Thought about some of the time, Thought about often), options range Likert 4–6 points scoring, and 1 reverse scoring question. Cronbach’s *α* = 0.843. The Cronbach’s coefficient for the present study was 0.843 (total score).

#### Index of General Affect

Campbell’s Index of General Affect Scale was a tool to describe the emotional state of oneself over a while through positive and negative emotions (Campbell, 1976), it’s an absolute emotional state (Ash, 2000). There are eight items on the scale, to ask our respondents to react to a series of paired adjectives, describing their lives in positive or negative terms, presented in the semantic differential format. Thus, they were asked to describe their lives in general as falling at a point they chose in the space between interesting and boring, enjoyable and miserable, lonely and friendly, rewarding and disappointing, and the like from 1 to 7. The Cronbach’s coefficient for the present study was 0.961 (total score).

#### Generalized Anxiety Disorder-7

A Chinese version of the Generalized Anxiety Disorder-7 (GAD-7) scale was used to assess the subject’s anxiety symptoms. The GAD-7 has been previously used in Chinese populations and was found to have good reliability (Cronbach *α* = 0.90; Cronbach *α* = 0.90; Tong et al., 2016). In our study, seven items assess the frequency of anxiety symptoms over the past 2 weeks on a four-point Likert scale ranging from 0 (never) to 3 (nearly every day). The total score of GAD-7 ranged from 0 to 21, with increasing scores indicating more severity resulting from anxiety (Spitzer et al., 2006). The Cronbach’s coefficient for the present study was 0.956 (total score).

#### Resilience

The Connor-Davidson resilience scale (CD-RISC; Connor and Davidson, 2003) was used to evaluate the resilience in the present study. The scale was developed based on concepts of hardiness, adaptation, and stress endurance and validated in diverse samples. Initial factor analyses identified five factors: (1) notion of personal competence, high standards and tenacity, (2) trust in one’s instincts, tolerance of negative affect, and strengthening effects of stress, (3) positive acceptance of change, and secure relationships, (4) control, and (5) spiritual influences, and the Chinese version was tested by Yu and Zhang (2007). The scale has a total of 25 items and uses a five-point scoring, from “0 to 4” to indicate “not at all to almost always.” The Cronbach’s coefficient for the present study was 0.987 (total score).

## Statistics

SPSS 24.0 was used for descriptive statistics, and the MATRIX macro compiled by Hayes et al. is used for inferential statistics (Hayes, 2013). Hypothesis 2 predicts that PRCP will positively influence GAD while hypotheses 3 predict that IGA will mediate the involvement PRCP GAD link. To test hypotheses 2 and 3, Preacher and Hayes’ (2008) mediation analysis, i.e., PROCESS Macro (model 5) was used. The specific steps are as follows: First, conduct common method deviation analysis; Second, descriptive analysis and Pearson correlation analysis of main variables; Third, use the PROCESS plug-in, select the independent variable, mediating variable, moderate variable, and dependent variable into the corresponding option box, in turn, select model 5, set the sample size to 5,000, select the nonparametric percentile bootstrap method for deviation correction, the confidence level of the confidence interval is 95%, and the grouping condition is mean and mean plus or minus a standard deviation.

### Common Method Deviation Test

The Harman single factor method was used to detect the common method deviation. The exploratory factor analysis results of 49 items show that there are seven factors with eigenvalues higher than 1, and the variance interpretation rate of the first factor is 46.16% (<50%), indicating that there is no serious common method deviation in this study (Podsakoff et al., 2003; Malhotra et al., 2006).

## Results

### Descriptive Statistics and Correlations Among PRCP, IGA, Resilience, and GAD

Most of the correlations between the variables were statistically significant. The correlation coefficients had different signs depending on particular variables. The PRCP was positively associated with GAD, and negatively associated with IGA and resilience. The IGA was negatively related to GAD but positively related to resilience. The GAD was negatively related to resilience. The correlation between gender, age, marital status, PRCP, IGA, resilience, and GAD were analyzed. There is a significant correlation between demographic variables and other variables except for marital status with IGA and resilience. Therefore, gender, age, and marital status will be included in the control variables. See Table 1 for details. H1 was verified.

**Table 1:** Intercorrelations and descriptive statistics for gender, marital status, age, PRCP, IGA, GAD, and resilience (*N* = 522).

	*M*	*SD*	1	2	3	4	5	6
1. Gender			1					
2. Marital status			0.014	1				
3. Age	21.970	2.257	0.035	−0.206^***^	1			
4. PRCP	21.730	6.890	0.125^**^	0.019	−0.169^***^	1		
5. IGA	41.243	11.530	−0.276^***^	−0.143^**^	0.097^*^	−0.252^***^	1	
6. GAD	4.384	3.743	0.245^***^	0.104^*^	−0.108^*^	0.267^***^	−0.637^***^	1
7. Resilience	69.470	20.803	−0.186^***^	−0.083	0.158^***^	−0.241^***^	0.612^***^	−0.473^***^

* *p* < 0.05;

** *p* < 0.01;

*** *p* < 0.001.

### Conditional Process Analysis

The Bootstrap mediation effect test method was used to test concerning the conditional process model (Model 5) proposed by Hayes. The results were shown in Tables 2, 3.

**Table 2:** Mediation between different levels of resilience to GAD.

	*SD*	*B*	*SE*	LLCL	ULCL
Direct	−1	0.219	0.047	0.127	0.312
	0	0.110	0.033	0.042	0.178
Indirect	1	−0.000	0.046	−0.090	0.089
		0.108	0.025	0.061	0.158

**Table 3:** Effect of the conditional process model.

	IGA				GAD			
	*B*	*SE*	*p*	95%CI	*B*	*SE*	*p*	95%CI
Common	1.138	0.556	<0.05	[0.046, 2.230]	0.042	0.453	0.924	[−0.847, 0.932]
Age	0.019	0.019	0.301	[−0.017, 0.056]	−0.014	0.015	0.352	[−0.044, 0.016]
Gender	−0.397	0.065	<0.001	[−0.526, −0.269]	0.092	0.055	0.093	[−0.015, 0.200]
Marital status	−0.456	0.150	<0.01	[−0.751, −0.162]	0.043	0.122	0.722	[−0.196, 0.283]
PRCP	−0.211	0.042	<0.001	[−0.292, −0.129]	0.110	0.035	<0.01	[0.041, 0.178]
IGA					−0.513	0.043	<0.001	[−0.597, −0.428]
Resilience					−0.122	0.042	<0.004	[−0.204, −0.038]
Int					−0.110	0.031	<0.001	[−0.170,-0.049]
*R*^2^ = 0.144					*R*^2^ = 0.447			

**Figure 2:**
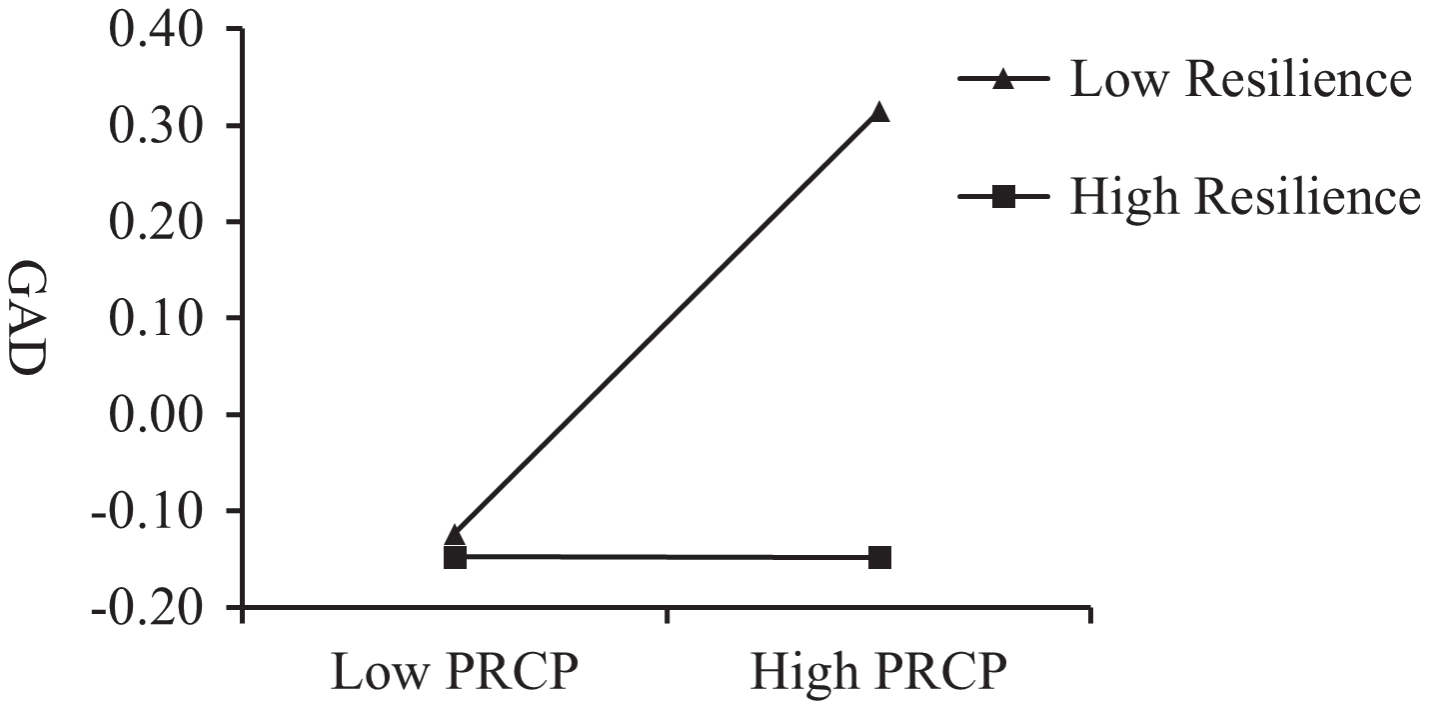
The association between PRCP and GAD is moderated by resilience.

Resilience score was divided according to *M*, *M* + *SD*, and *M*−*SD* and three elastic levels were obtained: medium, low and high. The direct impact of PRCP on GAD under different elastic levels was analyzed. As shown in Table 2 and Figure 2, it is found that under the high level of resilience, the direct effect of PRCP on GAD is not significant, and only under the medium and low level of resilience, the direct effect of PRCP on GAD is significant. The confidence intervals of 95% CI were [0.127, 0.312], [0.042, 0.177], and [−0.090, 0.089] at −1, 0 and +1 SD, respectively. The smaller the slope, the higher the impact of PRCP on GAD. H2 was verified.

As shown in Table 3, the indirect effect is 0.108, and the confidence interval 95% *CI* = [. 061, 0.158] does not include 0, indicating that the mediating effect is significant. It shows that PRCP has a positive predictive effect on anxiety GAD, and the IGA plays as a mediating variable between PRCP and IGA. H3 was verified.

## Discussion

The purpose of this study is to explore the impact of epidemic risk perception on anxiety after major public health events become normalized. Evaluating and controlling the mental state after public health events can effectively reduce the resulting diseases to be minimized (Sharot, 2011). We are mainly concerned about the 18–25-year-old emerging adults because most of them have not obtained a stable professional environment and spouse support. In the face of major public events, the lack of coping ability of young adults may bring corresponding mental health problems. It is found that the effects of PRCP on GAD will be partially mediated by the IGA, and emerging adults with lower resilience after they are perceiving the risk, they are more likely to be anxious.

Firstly, it is similar to previous studies our research results confirm the H1 that the GAD of emerging adults is significantly related to the PRCP (Oyetunji et al., 2021; Yin et al., 2021). For previous studies that do not support this result (Musche et al., 2021), we introduced resilience as a moderator variable to test. The final results support our H2 that resilience moderates the effect PRCP has on GAD. The direct effect of PRCP on GAD is not significant for people with a high level of resilience, but significant for people with a low level of resilience. From the perspective of resilience process theory, people with a high level of resilience have stronger dispositional resilience and are more likely to perceived more social support (Montpetit et al., 2010). In addition to social support, there is also the impact of individual ability. Dratva studied the relationship between College Students’ risk perception and GAD at Swiss university, shows that the GAD level of health students on campus is significantly lower (Dratva et al., 2020), expertise and experience on infection risks and health may have played a role. Resilience moderate the effect PRCP has on GAD explains the contradictions of previous studies. The research on diabetes patients is special for disease experience. From the perspective of Kumpfer and Bluth (2004) resilience framework theory, previous experience will make their resilience level higher. Therefore, even if their risk perception is high, there is no difference in anxiety under the effect of resilience. It helps researchers to understand the process mechanism of resilience and the mechanism of GAD caused by our perception in the face of stressful events. Therefore, in the specific work, we should pay attention to improving the level of resilience. The three-factor structure of resilience, social support, and perceived social support are key elements, as well as relevant health knowledge (Dratva et al., 2020), better education, and economic prosperity (Ruscio et al., 2017), etc. These factors can effectively reduce the incidence of GAD. For COVID-19, people know that they can have vaccines and medical care can be guaranteed (Jaspal and Breakwell, 2021), which are powerful and very effective channels to enhance resilience and reduce GAD.

For people with higher resilience, there is no significant correlation between their PRCP and GAD, combined with GAD’s emotional disorder theory, we know that GAD symptoms are mainly caused by emotional disorders (Mennin et al., 2007). Like Watson’s research showing emotional disorder strong and consistent associations with GAD (Watson and Naragon-Gainey, 2014). Therefore, PRCP may affect GAD through the emotional state. Therefore, the third hypothesis is that the IGA, which contains both positive and negative emotional state index scores, plays a meditating role in PRCP. The results of the current study support H3. That PRCP can lead to a decline in our IGA, which will slide from positive to negative. In this sliding process, the GAD symptoms will also increase as a result of the change of emotional state, which is consistent with previous studies (Ning et al., 2020; Mohammadkhani et al., 2021). Therefore, in the specific work, if we can pay attention to the scientific and rationality of risk publicity and improve the IGA, we can effectively block the formation path of GAD from PRCP and help to reduce the occurrence of GAD.

The conditional process model of H2 and H3 in this study shows that improving the level of residents’ resilience, paying attention to the active publicity in the process of epidemic risk, and regulating residents’ emotions can help to reduce GAD.

This study confirms two very effective hypotheses, but there are also some limitations. The moderator variables of the mediating path have not been explored clearly. The subject of this study is aimed at emerging adults. This study used a cross-sectional data set, which is liable as far as behavioral analysis is concerned. Such data cannot be used to explain cause and effect explicitly (Maxwell et al., 2011). It is the age of identity explorations, the age of instability (Arnett, 2007). However, for teenagers and adult groups, whether their GAD will be affected by the same conditional process model needs further research and discussion.

## Conclusion

In the face of major public health events, people will feel very anxious. It can be seen from the conditional process obtained, the higher PRCP, the more GAD. On the other hand, if we have enough resilience, such as enough medical knowledge or perceived support from society and family, PRCP will no longer have an impact on GAD. In the face of public health events, it may be wise to give priority to dealing with emotions. Intervention against negative emotions or improve positive emotion can effectively block the path of PRCP affecting GAD. This study further complements our understanding of the conditional process of GAD under COVID-19 risk.

## Data Availability Statement

The raw data supporting the conclusions of this article will be made available by the authors, without undue reservation.

## Ethics Statement

Ethical review and approval was not required for the study on human participants in accordance with the local legislation and institutional requirements. The patients/participants provided their written informed consent to participate in this study.

## Author Contributions

BW contributed to conception and design of the study and wrote sections of the manuscript. HF organized the database, performed the statistical analysis, and wrote the first draft of the manuscript. All authors contributed to the article and approved the submitted version.

## Funding

This work was supported by the Research Foundation of Student Education Management and Reform Research Project funded by Southwest University of Science and Technology (19sxb118), the Research & Instruction Center of Mianyang Adolescents’ Psychological Growth (SCWCN2019YB14), the Longshan Talents Plan of Southwest University of Science and Technology (18LSX610), the Psycho-social Service and Crisis Intervention Research Center of Southwest University of Science and Technology (108102), the Institute of Psychology, CAS, (GJ202003).

## Conflict of Interest

The authors declare that this review was conducted in the absence of any commercial or financial relationships that could be construed as a potential conflict of interest.

## Publisher’s Note

All claims expressed in this article are solely those of the authors and do not necessarily represent those of their affiliated organizations, or those of the publisher, the editors and the reviewers. Any product that may be evaluated in this article, or claim that may be made by its manufacturer, is not guaranteed or endorsed by the publisher.
